# Long-term follow-up for monovision surgery by Implantable Collamer Lens V4c implantation for myopia correction in early presbyopia

**DOI:** 10.1007/s00417-021-05545-x

**Published:** 2022-02-07

**Authors:** Yuhao Ye, Jing Zhao, Zhe Zhang, Lingling Niu, Wanru Shi, Xiaoying Wang, Xingtao Zhou

**Affiliations:** 1 Department of Ophthalmology and Optometry, Eye & ENT Hospital, Fudan University, No. 19 Baoqing Road, Shanghai, 200031 China; 2 NHC Key Laboratory of Myopia (Fudan University), Key Laboratory of Myopia, Chinese Academy of Medical Sciences, Shanghai, China; 3 Shanghai Research Center of Ophthalmology and Optometry, Shanghai, China

**Keywords:** Implantable collamer lens, Monovision, Myopia, Presbyopia, Safety, Efficacy

## Abstract

**Purpose:**

To investigate the long-term safety and efficacy of monovision surgery using implantable collamer lens V4c (ICL V4c) implantation in myopic patients with early presbyopia.

**Setting:**

Eye and ENT Hospital of Fudan University, Shanghai, China.

**Design:**

Prospective case series study.

**Methods:**

This study included 64 eyes of 32 patients with early presbyopia, who underwent bilateral ICL V4c implantation for myopia correction. Parameters, including mean spherical equivalent (SE), uncorrected distance visual acuity, corrected distance visual acuity, intraocular pressure, endothelial cell density, presbyopic add power, visual acuity (logMAR) of dominant eyes (D-eye), nondominant (nD-eye) eyes, and both eyes (Bi) at 0.4 m, 0.8 m, and 5 m were recorded at the last follow-up.

**Results:**

All surgeries were uneventful. At the last follow-up, the safety indices were 1.23 ± 0.18 (D-eyes) and 1.21 ± 0.18 (nD-eyes) (*p* > 0.05); the efficacy indices were 0.95 ± 0.27 (D-eyes) and 0.92 ± 0.28 (nD-eyes) (*p* < 0.05), the SE was -0.62 ± 0.47 D (D-eyes); and − 1.21 ± 0.78D (nD-eyes) (*p* < 0.05), presbyopic add power was 1.31 ± 0.58 D. The visual acuity (logMAR) of D-eyes, nD-eyes, and binocular (Bi) at 5.0 m were: 0.06 ± 0.15 (D-eye), 0.21 ± 0.18 (nD-eye), (*p* < 0.01), and 0.04 ± 0.13 (Bi); 0.8 m: 0.03 ± 0.18 (D-eye), 0.08 ± 0.16 (nD-eye), (*p* > 0.05), and − 0.02 ± 0.11 (Bi); 0.4 m: 0.08 ± 0.09 (D-eye), − 0.02 ± 0.08 (nD-eye), (*p* < 0.001), and − 0.03 ± 0.09 (Bi). Subjects were very satisfied or felt excellent with their visual acuity at near (81.25%) and far distances (87.50%), respectively (versus preoperative, *p* < 0.001).

**Conclusion:**

Monovision surgery using ICL V4c implantation is safe and practicable for correction of myopes with presbyopia, with long-term efficacy at near and far distances and patient satisfaction.

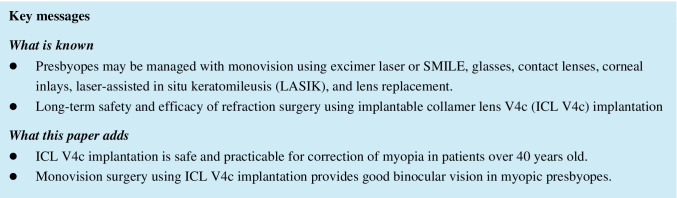

## Introduction

As the population ages, an increasing number of people, which was estimated to be 2.1 billion worldwide by 2020 [1], is affected by presbyopia, the age-related loss of accommodation. Presbyopes may be managed using monovision [2], in which the dominant eye (D-eye) may be fully corrected for distance vision, and the nondominant eye (nD-eye) may be undercorrected for near vision, thus producing monocular blur. Earlier, methods such as excimer laser or SMILE were used to correct myopic presbyopia [3]. In addition, glasses, contact lenses, corneal inlays, laser-assisted in situ keratomileusis (LASIK), and lens replacement showed the clinical value of monovision for presbyopia. In patients with high myopia, corneal implants [1] and corneal laser surgery [3] may induce high-order aberrations and reduce contrast sensitivity. Refractive lens replacement can cause loss of accommodation and increase the risk of retinal detachment.

Visian ICL (ICL, STAAR Surgical, Nidau, Switzerland) is a phakic chamber intraocular lens (pIOL) with good performance in the correction of different degrees of myopia [4]. Previous studies have shown that ICL implantation is safe and effective in correcting hyperopia and hyperopic astigmatism [5]. With the clinical application of ICL with a central hole (hole ICL), or ICL V4c, the misgivings regarding possible effect of ICL on cataract formation in the elderly population have been dispelled as it improves aqueous humor circulation [6, 7]. ICL V4c is also safe for the correction of ametropia in myopic people aged 40 years or above [8]. ICL V4c implantation is reversible; therefore, its application in monocular refractive surgery is worthy of further study. However, to the best of our knowledge, no long-term research has been conducted to observe outcomes of ICL V4c implantation in myopic presbyopes.

Therefore, this study aimed to assess the safety, efficacy, and predictability of monovision surgery by ICL V4c implantation in myopic presbyopes and provide a theoretical basis for this treatment modality. The visual acuity (VA) of the D-eye, nD-eye eye, and binocular (Bi) was evaluated at near and far distances.

## Materials and methods

### Subjects

This prospective observational consecutive case series included 64 eyes of 32 patients with early presbyopia (male/female: 12/20, average age: 43.50 ± 2.62 years old, range 40 to 50 years) who received ICL V4c implantation to correct myopia or myopia with astigmatism at the Eye and ENT Hospital of Fudan University, Shanghai, China, between April 2016 and December 2017. This study was conducted in accordance with the Declaration of Helsinki and was approved by the Ethics Committee of the Eye and ENT Hospital of Fudan University. All procedures were performed after obtaining written informed consent from the patients. The inclusion criteria were as follows: age ≥ 40 years, stable refractive error (increase of < 0.5D/year) within 2 years, no use of soft contact lens for ≥ 2 weeks, no use of rigid gas permeable contact lens for ≥ 4 weeks, and presbyopic add power ≥  + 0.50 D. The exclusion criteria were as follows: preoperative corrected distance VA (CDVA) (LogMAR)  > 0.15, anterior chamber depth (ACD) < 2.8 mm or endothelial cell density (ECD) < 2000cell/mm^2^, history of ocular inflammation or trauma, lens opacity, glaucoma, previous eye surgery, other diseases of the eye or systemic diseases.

### Preoperative examinations

Spherical equivalent (SE), CDVA, and uncorrected-distant VA (UDVA) were measured by an experienced ophthalmologist using a phoropter (RT-5100, Nidek Technologies, Japan). Presbyopic add power was measured with a Fusion Cross-Cylinder (FCC) at the distance of 33 cm, and the D-eyes and nD-eyes were determined by the card-hole method preoperatively. Slit lamp examination and fundus examination were completed after pupillary dilation. Intraocular pressure (IOP) was measured using a Canon Full Auto Tonometer TX-F (Canon, Inc., Tokyo, Japan); ECD by SP-2000P (Topcon Corporation, Kyoto, Japan); corneal thickness, white to white (WTW); and ACD, angle to angle (ATA) and anterior chamber volume (ACV) by Pentacam HR (Oculus Optikgerate Wetzlar, Wetzlar, Germany). WTW was also measured by IOL Master 700 (Carl Zeiss AG, Germany).

### Lens power and size calculation

Lenses were transparent in patients in this study and they selected ICL V4c implantation to retain their natural accommodation. The ICL power was determined using an online calculator provided by the manufacturer (STAAR Surgical). The D-eyes were targeted for approximately − 0.75 to 0 D (− 18.00 D was selected as the target refraction correction at the spectacle plane for eyes <  − 18.00 D) and the nD-eyes for around − 2.25 to − 0.50 D, according to the presbyopic add power of each patient. In the patient with D-eyes <  − 18.00D, − 18.00D was selected as the target refraction correction, resulting in r*e*sidual myopic diopters as the target refraction in some D-eyes, which was on trial in frame glasses preoperatively and accepted by the patients. The ICL size calculation was based on the horizontal WTW, ACD, and ATA distances. We applied an adjustment to the WTW value from Pentacam and referred value from the IOLmaster measurements for ICL sizing.

### Implantable collamer lens surgical procedure

All ICL V4c implantation surgeries were successfully performed by two experienced doctors (Dr. Xingtao Zhou and Dr. Xiaoying Wang). The patients were administered antibiotic eye drops 4 times a day for 3 days before the operation. Performed under topical anesthesia, the ICL V4c were implanted into the anterior chamber which was pre-injected with a viscoelastic agent through the lateral corneal incision. Thereafter, the ICL was adjusted using the manipulator, and the viscoelastic agent was replaced with a balanced salt solution. The detailed steps have been described previously [9]. Postoperative antibiotic eye drops and steroid eye drops were administered 4 times a day for 2 weeks and tapered gradually.

### Postoperative examinations

At 1 month and 3 months postoperatively, and at the last follow-up, data on the UDVA, SE, CDVA, IOP, ECD (except at 1 month postoperatively), WTW, ACD, and ACV, was recorded. Presbyopic add power, CDVA, and UDVA (logMAR) of D-eyes, nD-eyes, and both eyes at 0.4 m, 0.8 m, and 5 m were recorded at the last follow-up. Measurements were performed under mesopic conditions using two trial frames [14] and two tumbling E charts (VSK-VC-J 0.4 m/0.8 m, Wehen Vision, China) for the monocular and binocular VA at the distance of 0.4 m and 0.8 m, respectively, and a phoropter was used for monocular and binocular distant VA at 5 m. The safety index (SI) was defined as postoperative CDVA over preoperative CDVA, efficacy index (EI) was defined as the postoperative UDVA over preoperative CDVA, predictability was defined as the comparison between postoperative and target SE, and stability was defined as the SE change at long-term follow-up. The subjective satisfaction of VA at near and far distances was recorded preoperatively, 3 months postoperatively, and at the last follow-up (scores 1–5: 1, very dissatisfied; 2, dissatisfied; 3, satisfactory; 4, highly satisfactory; and 5, feel excellent).

### Statistical analysis

All statistical analyses were performed using the Statistical Package for the Social Sciences version 25.0. (SPSS, Inc., Chicago, IL, USA). The results are expressed as mean ± standard deviation. Normality of the data was checked using the Kolmogorov–Smirnov test. One-way/repeated ANOVA was used to compare the pre- and post-treatment normally distributed data, and Wilcoxon signed-rank test was used for non-normally distributed data. The Pearson correlation coefficient was employed to analyze the correlation between the add power or changes in such and other parameters. Differences were considered statistically significant at *p* < 0.05.

## Results

All patients successfully and uneventfully completed the last follow-up at 43.19 ± 7.06 months (range 33 to 58 months) postoperatively. Table 1 provides an overview of the preoperative patient demographics. All types of data loss were < 5%.

**Table 1:** Preoperative patient demographics

Characteristic	Mean ± SD	Range
Age (years)	43.50 ± 2.61	[40, 50]
Gender (male/female)	12/20	
Axial length (mm)	28.86 ± 2.35	[24.99, 32.96]
Refraction sphere (D)	− 11.72 ± 3.62	[− 19.50, − 4.00]
Refraction cylinder (D)	− 1.32 ± 0.98	[− 3.50, 0]
Spherical equivalent, SE (D)	− 12.37 ± 3.67	[− 20.00, − 5.63]
Dominant eye (OD/OS)	15/17	
K-flat (D)	42.99 ± 1.67	[39.50, 46.90]
K-steep (D)	44.49 ± 1.90	[40.50, 47.30]
CT (mm)	0.52 ± 0.33	[0.460, 0.590]
ATA, vertical (mm)	12.41 ± 0.60	[11.19, 14.80]
ATA, horizontal (mm)	11.91 ± 0.54	[10.94, 14.17]
WTW (mm)	11.64 ± 0.40	[10.90, 12.80]

*SE*, spherical equivalent; *IOP*, intraocular pressure; *CDVA*, corrected-distant visual acuity; *CT*, corneal thickness; *ATA*, angle to angle distance; *WTW*, white to white distance

### Safety and efficacy

The safety indices for all eyes at 1 month, 3 months postoperatively, and at the last follow-up were 1.17 ± 0.17, 1.17 ± 0.21, and 1.22 ± 0.18, respectively. The corresponding efficacy indices were 1.03 ± 0.25, 1.04 ± 0.27, 0.85 ± 0.29, respectively. The safety and efficacy indices of the D-eyes and the nD-eyes are listed in Table 2.

**Table 2:** The clinical parameters of the dominant eyes or nondominant eyes before and after the implantable collamer Lens V4c implantation

Characteristic	D- or nD-eye	Preoperative	1-mon follow-up	3-mon follow-up	Last follow-up
UDVA (Logmar)	D-eye nD-eye	NA	− 0.03 ± 0.08# 0.08 ± 0.13#	− 0.02 ± 0.10* 0.06 ± 0.14*	0.06 ± 0.15*▵▴ 0.20 ± 0.19*▵▴
SE (D)	D-eye nD-eye	− 12.00 ± 3.66# − 12.74 ± 3.70#	− 0.14 ± 0.35#* − 0.69 ± 0.64#*	− 0.19 ± 0.15#* − 0.72 ± 0.73#*	− 0.62 ± 0.47#*▵▴ − 1.21 ± 0.78#*▵▴
CDVA (Logmar)	D-eye nD-eye	0.02 ± 0.08# 0.03 ± 0.09#	− 0.05 ± 0.06* − 0.03 ± 0.06*	− 0.05 ± 0.07* − 0.03 ± 0.07*	− 0.06 ± 0.47* − 0.06 ± 0.08*
Safety indices	D-eye nD-eye	NA	1.19 ± 0.17 1.15 ± 0.18	1.19 ± 0.18 1.16 ± 0.24	1.23 ± 0.18 1.21 ± 0.18
Efficacy indices	D-eye nD-eye	NA	1.14 ± 0.19# 0.92 ± 0.26#	1.12 ± 0.24# 0.96 ± 0.26#	0.95 ± 0.27#▵▴ 0.92 ± 0.28#▵▴

*D-eye*, dominant eye; *nD-eye*, nondominant eye; *UDVA,* uncorrected distance visual acuity; *SE*, spherical equivalent; *CDVA*, corrected distance visual acuity

^*^Versus preoperative, *p* < 0.05

▵The last follow-up versus the 1-month follow-up, *p* < 0.05

▴The last follow-up versus the 3-month follow-up, *p* < 0.05

^#^Dominant eye versus nondominant eye, *p* < 0.05

The percentage of D-eyes with UDVA ≥ 20/16 (Snellen line) was 62.50%, 56.25%, and 25.13% at 1 month, 3 months postoperatively, and at the last follow-up, respectively. UDVA ≥ (20/20) was 81.25%, 75.00%, and 62.50%, 1 month, 3 months postoperatively, and at the last follow-up, respectively. UDVA ≥ (20/40) was 100.00% at all the three follow-up time points. At the last follow-up, 62.50% of the D-eyes had a UDVA ≥ preoperative CDVA.

The percentage of the nD-eyes with UDVA ≥ (20/16) (Snellen line) was 18.75%, 28.13%, and 12.50% at 1 month, 3 months postoperatively, and at the last follow-up respectively. UDVA ≥ (20/20) was 43.75%, 50.00%, and 25.00% at 1 month, 3 months postoperatively, and at the last follow-up, respectively. UDVA ≥ (20/40) was 100.00%, 100.00%, and 87.50% at 1 month, 3 months postoperatively, and at the last follow-up respectively. At the last follow-up, 21.88% of the nD-eyes had a UDVA ≥ preoperative CDVA.

**Fig. 1:**
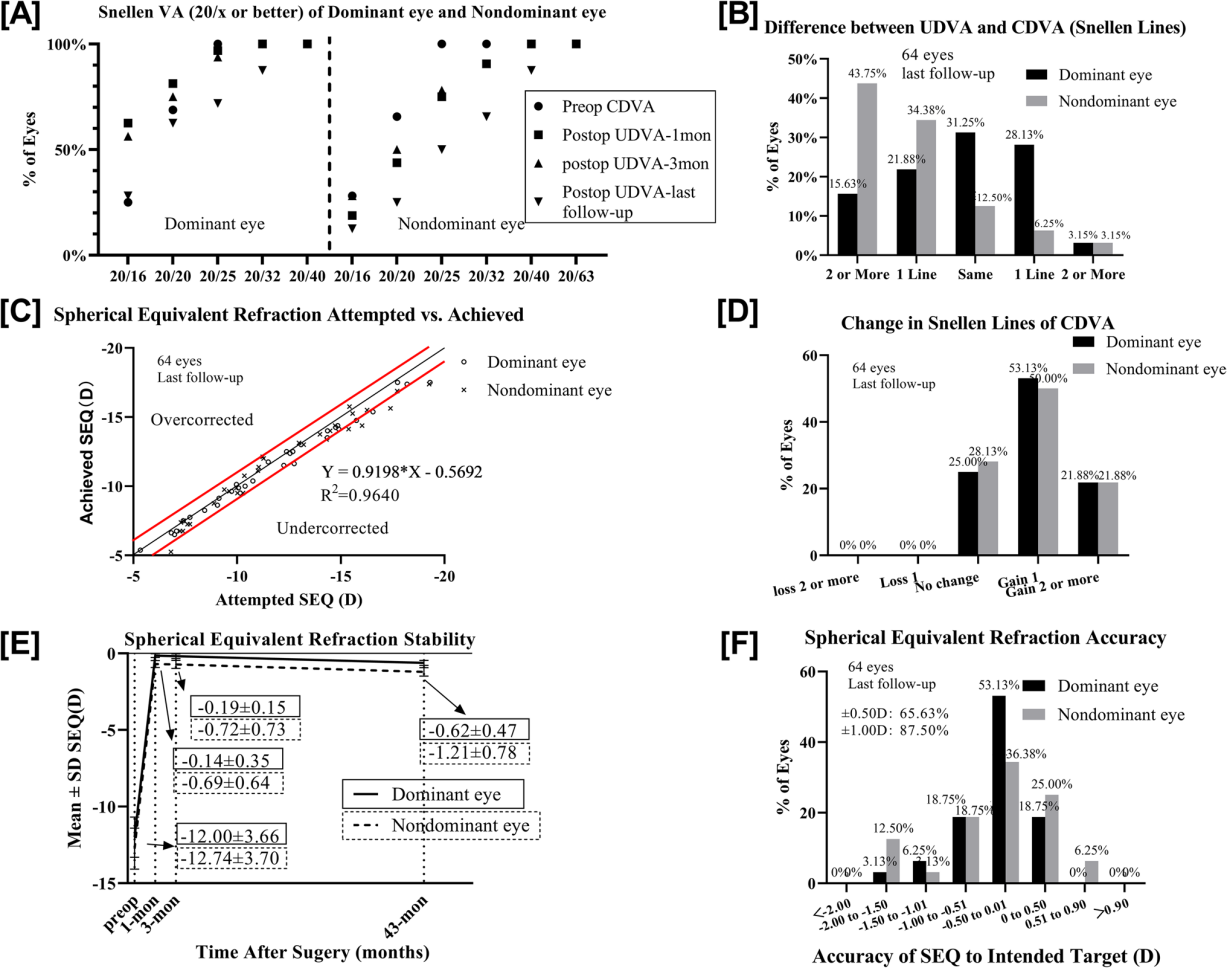
Clinical outcomes of 64 eyes (32 dominant eyes and 32 nondominant eyes) with myopia at the last follow-up after the implantation of Implantable Collamer Lens V4c

The percentage of D-eyes and nD-eyes that had an increased CDVA (Snellen lines) for ≥ 2 lines were both 21.88%; for ≥ 1 line were 53.13% and 50.00%, respectively; and for decreased CDVA were 0% and 0%, respectively (Snellen lines) (Fig. 1A, B, and C).

### Monovision

**Fig. 2:**
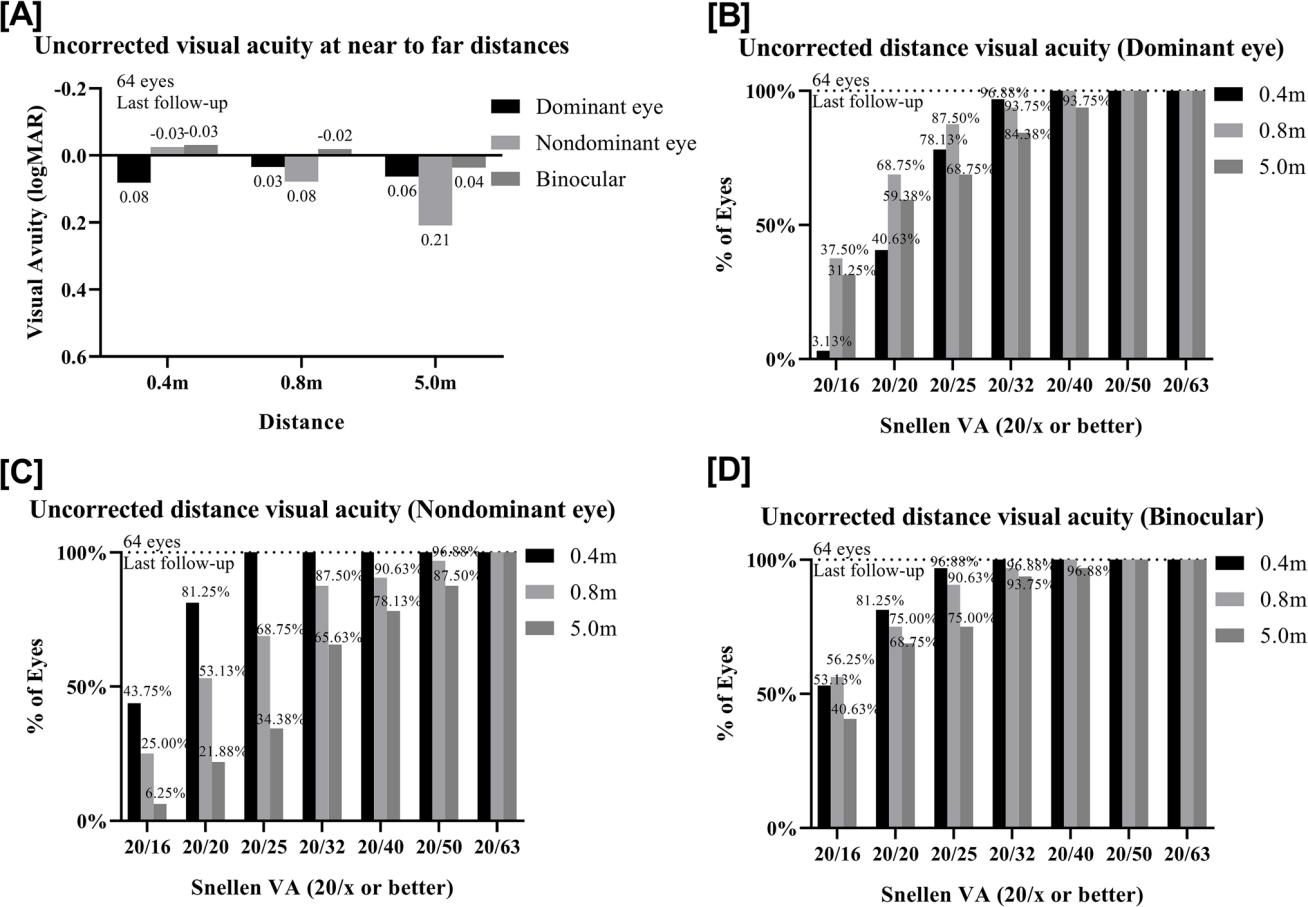
Uncorrected distance visual acuity at near and far distances at 43 months after hole implantable collamer lens V4c (ICL V4c) implantation. **A** Last follow-up UDVA (logMAR) of Dominant eyes, nondominant eyes and binocular. **B** Last follow-up UDVA (Snellen) of the dominant eyes. **C** Last follow-up UDVA (Snellen) of the nondominant eyes. **D** Last follow-up binocular UDVA (Snellen)

Presbyopic add power increased by 0.17 D/year from 0.69 ± 0.40 D preoperatively to 1.31 ± 0.58 D at the last follow-up. Five patients had a dominant eye switch. The UDVA, SE, and CDVA values are presented in Table 2. There was no significant difference between the presbyopic add power and the absolute value of SE for the nD-eyes at the last follow-up (*p* > 0.05). Figure 2A provides an overview of the VA of the D-eye, nD-eye, and binocular at near to far distances. Figure 2B, C, and D compare their performances in Snellen VA.

### Predictability and stability

At 1 month, 3 months postoperatively and the last follow-up, 81.25%, 78.13%, and 71.88% of the D-eyes were within the range of ± 0.50 D and 100%, 96.88%, and 90.63% were within the range of ± 1.00 D, respectively.

At 1 month, 3 months postoperatively and the last follow-up, 68.75%, 59.38%, and 59.38% of the nD-eyes were within the range of ± 0.50 D and 96.88%, 85.70%, and 84.38% were within the range of ± 1.00 D, respectively.

### Anterior segment parameters

A decrease of 0.23 ± 0.25 mm (6.88 ± 7.12%) was observed in the ACD at the last follow-up as compared with that at the preoperative level. The corresponding values were 71.49 ± 18.56 mm^3^ (36.86% ± 6.17%), 15.42 ± 4.84° (39.97% ± 9.75%) for ACV, and ACA (anterior chamber angle), respectively. In comparison with the values at 3 months postoperatively, the vault decreased by 45.78 ± 95.90 μm (8.92% ± 19.75%) at the last follow-up (Table 3). Furthermore, 3.13% of eyes had their vault within the range of 170–200 μm, 89.06% within the range of 200–750 μm, and 7.81% > 750 μm. Cataract and high IOP (> 21 mmHg) were not observed in any of the eyes.

**Table 3:** The clinical parameters and biometric values of the eyes before and after the implantable collamer lens V4C implantation

Characteristic Range [min, max]	Preoperative	1-mo follow-up	3-mo follow-up	Last follow-up
CDVA (Logmar) Range [min, max]	0.02 ± 0.08 [− 0.08, 0.15]	− 0.04 ± 0.06* [− 0.18, 0.10]	− 0.04 ± 0.07* [− 0.18, 0.10]	− 0.06 ± 0.08* [− 0.18, 0.15]
Spherical equivalent, SE (D) Range [min, max]	12.37 ± 3.67 [− 20.00, − 5.63]	− 0.41 ± 0.58* [− 2.25, 0.50]	− 0.45 ± 0.65* [− 2.50, 1.00]	− 0.91 ± 0.70*▵▴ [− 2.63, 0.00]
Presbyopic add power (D) Range [min, max]	0.69 ± 0.41 [0.50, 2.25]	NA	NA	1.31 ± 0.58* [0.50, 2.25]
ACD (mm) Range [min, max]	3.21 ± 0.31 [2.78, 3.97]	3.00 ± 0.32* [2.02, 3.86]	3.00 ± 0.32* [2.02, 3.81]	2.99 ± 0.31* [2.05, 3.81]
IOP (mmHg) Range [min, max]	14.91 ± 2.67 [8.40, 20.10]	14.29 ± 2.06 [10.40, 18.80]	14.71 ± 2.38 [9.4, 19.20]	15.07 ± 2.65 [9.90, 20.90]
ACV (μl) Range [min, max]	192.54 ± 33.33 [121.00, 295.00]	113.58 ± 22.78* [72.00, 190.00]	114.08 ± 22.57* [77.00, 198.00]	121.14 ± 22.08* [78.00, 186.00]
ACA (°) Range [min, max]	37.98 ± 5.31 [27.00, 47.30]	22.48 ± 4.56* [13.20, 31.00]	22.04 ± 4.35* [14.00, 30.20]	22.63 ± 3.51* [16.00, 28.20]
ECD (cell/mm2) Range [min, max]	3008.84 ± 550.57 [2259.00, 4595.00]	NA	2760.61 ± 404.99* [2125.00, 3932.00]	2525.34 ± 248.84*▴ [2047.00, 3198.00]
Vault (μm) Range [min, max]	NA	546.88 ± 184.65 [200.00, 880.00]	537.66 ± 179.52 [200.00, 830.00]	491.88 ± 206.24 [170.00 ± 860.00]

*CDVA*. corrected distance visual acuity; *SE*. spherical equivalent; *ACD*, anterior chamber depth; *IOP*, intraocular pressure; *ACA*, anterior chamber angle; *ACV*, anterior chamber volume; *ECD*, endothelium cell density

^*^Versus [reoperative, *p* < 0.05

▵The last follow-up versus the 1-month follow-up, *p* < 0.05

▴The last follow-up versus the 3-month follow-up, *p* < 0.05

### Endothelial cell density

As shown in Table 3, the ECD decreased by 483.5 ± 609.14 cell/mm^2^ (13.63% ± 15.54%) at the last follow-up, and decreased by 134.93 cell/mm^2^ (3.80%) every year. ECD in none of the eyes decreased to < 2000cell/mm^2^.

### Subjective satisfaction

**Fig. 3:**
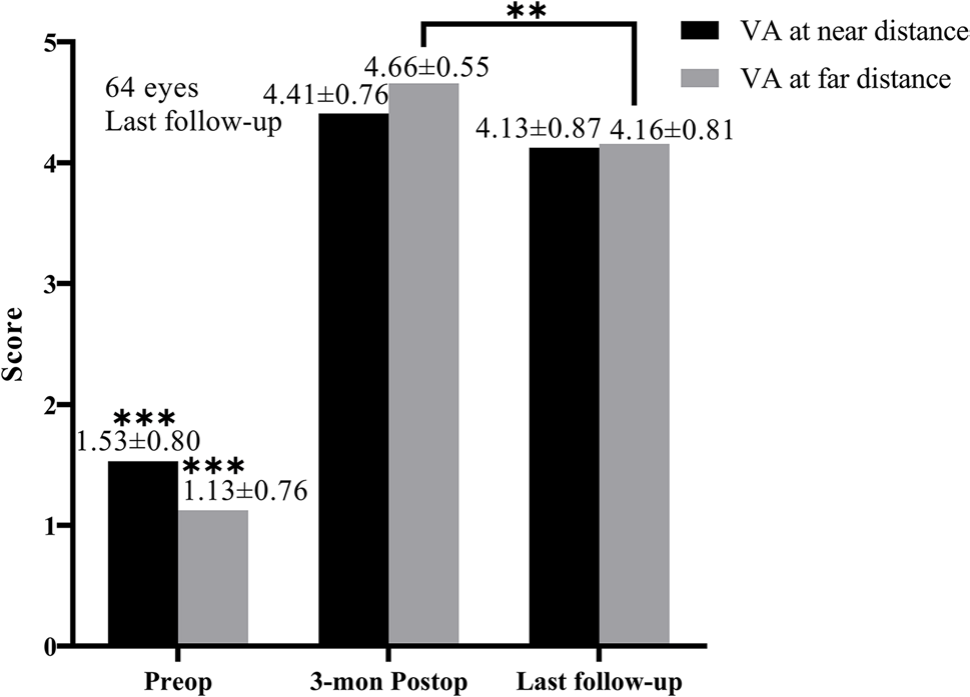
Subjective visual acuity satisfaction at near and far distances. ^**^p < 0.01, ^***^preop vs. postop, *p* < 0.001

The subjective satisfaction with near and far distances is shown in Fig. 3. Preoperatively, 3 months postoperatively, and at the last follow-up, 6.25%, 84.38%, and 81.25% participants, respectively, were very satisfied or felt excellent (subjective satisfaction scores of 4 or 5) with their VA at near distance, and 0%, 96.88%, and 87.50% participants, respectively, were very satisfied or felt excellent with their VA at far distance.

### Correlation analysis

There was no significant correlation between presbyopic add power and the anterior segment parameters (ACA, ACV, ACD, or vault), and there was also no significant correlation between the changes in presbyopic add power with the same parameters. The VA of the D-eye at 0.4 m showed a correlation with presbyopic add power at the preoperative level (*r* = 0.392, *p* < 0.05), the level at the last follow-up (*r* = 0.587, *p* < 0.001), and the difference of those two levels (*r* = 0.354, *p* < 0.05). The VA of the nD-eye at 5 m showed a correlation with presbyopic add power at the last follow-up (*r* = 0.469, *p* < 0.01). The binocular VA at both 0.4 m and 5 m distance showed a correlation with presbyopic add power at the last follow-up (*r* = 0.421, *p* < 0.05). There was no significant correlation between myopic shift (SE at the last follow-up minus that at 3 months postoperatively) and the decrease of vault (vault at the last follow-up minus that at 3 months postoperatively) (*r* =  − 0.094, *p* = 0.444).

## Discussion

Presbyopia is the most common refractive error due to age-related loss of accommodation, which impairs the ability to change the refractive power of the crystalline lens and to focus at different distances. Presbyopes may lose their ability to accommodate at the age of 50 years when the crystalline lens loses elasticity [10]. Monovision is a conventional and appealing choice for presbyopes with good visual quality and patient satisfaction [11].

In this study, the SI and EI at the 43 months follow-up were 1.22 ± 0.18 and 0.85 ± 0.29, respectively. The proportion of eyes with UDVA ≥ 20/20 reached 68.75%, VA ≥ 20/40 at 40 cm reached 100%, and the satisfaction rate (subjective satisfaction scores of 4 or 5) reached 81.25% (vision at near distance) and 87.50% (vision at far distance), similar to the results of previous studies. Studies on conventional refractive surgery using LASIK have demonstrated that 84.7% of eyes had UDVA ≥ 20/20 (Snellen lines) at 3 months postoperatively, 90.7% VA ≥ 20/40 at 0.4 m, and 86.7% overall satisfaction rate [12]. And such figures were 100% ≥ 20/32, 100% ≥ 20/40 at 0.33 m, and 86.7% of the overall satisfaction rate 1 year postoperatively using SMILE with 1.03 of the SI and 1.04 of the EI [13]. It was observed that the distant VA of the patients at the last follow-up in this study was slightly lower than that at 3 months postoperatively, as shown by the significant decrease in SE and EI. Myopia drift may be related to the high proportion of patients with ultra-high myopia and the further progression of myopia.

To date, research has tended to focus on monovision by laser corneal surgery rather than ICL implantation. Kamiya et al. (2017) demonstrated a binocular UDVA (< 0.01 logMAR) half a year after ICL V4c implantation at near and far distances (0.3, 0.5, 0.7, 1.0, 2.0, 3.0, and 5.0 m) [14]. The corresponding figures in this study were < 0.04 logMAR after ICL V4c implantation at 0.4, 0.8, and 5.0 m. The results show a better moderate distance VA than that at far distance in monovision, which agrees with the findings of Nitta et al., which showed a similar improvement in VA at moderate distance by contact lenses [15]. The presbyopic add power at the last follow-up and the difference in add power were related to the VA of the D-eye at a 0.4 m distance, in accordance with the impaired VA at near distance of D-eye in monovision. The presbyopic add power at the last follow-up was related to the VA of the nD-eye at a 5.0 m distance, which is in accordance with the monovision concept of intentional undercorrection of the nD-eye. These results provide the first evidence of the long-term safety and efficacy of ICL V4c implantation in presbyopic myopes, with additional information on monovision refractive surgery and the long-term observation of monovision by ICL V4c implantation.

There was no significant difference in the IOP at any time point, and the ECD decreased by 3.8% per year. Compared with 0.6% per year in healthy adults [16] and 0.93% per year in patients who underwent ICL V4c implantation [17], the higher rate in the present study may be related to the age of the subjects, as the enrolled subjects were over 40 years of age, while previous study mostly have tended to enroll younger subjects. We selected 3 months postoperatively as the follow-up time point as previous findings have disclosed a nonsignificant difference in ECD at this time point [17], which excluded the effect of surgical procedures on ECD. ACD, ACA, and ACV showed no significant difference at any postoperative time point, although they were significantly reduced compared with the preoperative level. All vaults were greater than 150 μm after surgery, which was considered the minimal safe value [18]. In those patients with larger decreases in vault, no significant myopic shift was observed at the end of follow-up, meaning the vault may not be the explanation behind the myopic shift.

The axial length change during the follow-ups period can provide a clue for the myopic shift. However, this study did not include enough data for statistical analysis of this. Further study is required to demonstrate the AL change in ultra-high myopes in patients over 40 years of age. The current study was unable to analyze contrast sensitivity or near stereoacuity. Five patients had their D-eye switched, which could be attributed to constant reading or prolonged close work. Individuals, such as programmers, civil servants, or clerks, may get used to seeing at a closer distance. Further research is recommended on why and how the D-eye switch occurs. To the best of our knowledge, there is yet to be any research concerning lens replacement by multifocal intraocular lenses (MIL). Further work is required to compare these two surgeries and to establish a method to evaluate MIL in patients after ICL-V4c implantation.

In conclusion, the present study provides additional evidence with respect to good binocular vision and long-term safety and efficacy of monovision surgery by ICL V4c implantation in presbyopic myopia.
